# Human Interleukin-1*β* Profile and Self-Reported Pain Monitoring Using Clear Aligners with or without Acceleration Techniques: A Case Report and Investigational Study

**DOI:** 10.1155/2022/8252696

**Published:** 2022-08-31

**Authors:** Selma Pascoal, Aline Gonçalves, Andreia Brandão, Duarte Rocha, Sofia Oliveira, Francisca Monteiro, Óscar Carvalho, Susana Coimbra, Teresa Pinho

**Affiliations:** ^1^UNIPRO—Oral Pathology and Rehabilitation Research Unit, University Institute of Health Sciences (IUCS), CESPU, Porto, Portugal; ^2^Center for Microelectromechanical Systems (CMEMS), University of Minho, Campus Azurém, Braga, Portugal; ^3^LABBELS—Associate Laboratory, Braga, Guimarães, Portugal; ^4^ICVS/3B's—PT Government Associate Laboratory, Braga, Guimarães, Portugal; ^5^UCIBIO, REQUIMTE, Laboratory of Biochemistry, Portugal; ^6^TOXRUN—Toxicology Research Unit, University Institute of Health Sciences, CESPU, CRL, Gandra, Portugal; ^7^IBMC—Instituto Biologia Molecular e Celular, I3S—Inst Inovação e Investigação em Saúde, Universidade Do Porto, Porto, Portugal

## Abstract

**Introduction:**

There is a growing demand for more aesthetic, comfortable, and faster orthodontic treatments, and clear aligners emerged as a solution to fulfill this need. However, the effectiveness of clear aligners to treat complex malocclusions is yet contentious. The use of acceleration methods could improve the efficacy of clear aligners by stimulating cells' mechanobiology through numerous pathways, but this hypothesis is still poorly explored.

**Objective:**

We aimed to monitor the release profile of an inflammatory marker-the interleukin-1*β*-and to evaluate its relationship with self-reported pain scores with and without the use of acceleration techniques during an orthodontic treatment requiring difficult tooth movements with clear aligners. *Case Report*. Here, we report a case of a 46-year-old female patient who presented functional and aesthetic complaints. Intraoral examination revealed a diminished overjet and overbite, rotation of teeth 45 and 24, absence of teeth 25, 35, and 36, buccolingual dislocation of tooth 21, a tendency to a Class III malocclusion, and a 2 mm left deviation of the lower midline. This study is divided into three stimulation phases: no stimulation, mechanical vibration stimulation, and photobiomodulation. Interleukin-1*β* levels in gingival crevicular fluid samples from the pressure side of six selected teeth were evaluated at four time points after the orthodontic treatment onset. Pain monitoring in those teeth was performed using a visual analogue scale at the same time points.

**Results:**

Interleukin-1*β* protein production peaked 24 h after treatment onset. Complex movements were associated with increased self-reported pain.

**Conclusion:**

Clear aligners show limitations in solving complex tooth movements, even when combined with acceleration. The development of customized and programmable stimulation microdevices integrated into “smart aligners,” which could be designed to specifically stimulate the direction of movement and stimulation parameters and could constitute a solution to optimize the orthodontic tooth movement with clear aligners.

## 1. Introduction

Unlike physiological tooth movement, orthodontic tooth movement (OTM) mechanically induces a biological response that disrupts the equilibrium of the dentofacial complex [1]. Recently, several histological studies on OTM have demonstrated that each tooth moves in the periodontal space, creating pressure and tension areas as a consequence of mechanical stress. The pressure maintained on the teeth is the fuze of its movement into a different position within the periodontal space, compressing in some areas while stretching it in others [2–5]. This leads to structural modifications of the periodontal ligament accompanied by alterations of the biochemical environment in the gingival crevicular fluid (GCF) [6]. Among others, the release of proinflammatory molecules is one of the primary biological responses induced by the OTM. In particular, interleukin-1*β* (IL-1*β*) is secreted by osteoclasts as an immediate response to mechanical stress during the initial stage of the OTM. The survival, fusion, and activation of osteoclasts correlate with the IL-1*β* levels, which also determine the amount of tooth movement as IL-1*β* regulates alveolar bone remodeling [2, 7, 8]. IL-1*β* levels can increase significantly between 1 and 24 h after the beginning of the OTM, peaking at 24 and 72 h [7]. Then, IL-1*β* release significantly decreases until 168 h to baseline levels. Importantly, the application of a second force can augment the IL-1*β* to higher peaks than before [2, 7]. In this sense, this biomarker works as a reliable proxy for the evaluation of the inflammatory response and bone formation induced by the OTM over time.

To shorten the treatment duration and limit the side effects of the OTM, different acceleration techniques have been investigated. Two noninvasive, painless, and effective stimulatory approaches to accelerate the OTM have stood out—photobiomodulation (PBM) and mechanical vibrations (MV) [9]. PBM is a light-based low-cost and noninvasive approach that involves the exposure of tissues to red or near-infrared light (NIR) to promote a therapeutic effect in multiple biomedical applications, such as cartilage regeneration and transcranial stimulation therapies against neurological disorders [10–12]. Wavelengths from 600 to 1200 nm are used, and photon absorption by hemoglobin and water is reduced, allowing it to reach deeper tissues and the alveolar bone. PBM stimulates the proliferation of osteoclasts, osteoblasts, and fibroblasts, thereby, affecting bone remodeling and accelerating tooth movement [13–15]. Similarly, recent literature suggests that MV presents numerous therapeutic abilities in different scientific fields [10, 16, 17], including orthodontics [18, 19]. Several hypotheses have been proposed to explain how MV enhances the rate of orthodontic tooth movement. It has been reported that MV may stimulate the differentiation of osteoclasts from hematopoietic cells by increasing blood flow. These signals may be mediated in response to direct effects on the cell membrane, changes in ion transport, activation of stretch-activated channels, activator changes in the attachments between skeletal bones and extracellular matrix, or modification of intracellular signals that regulate gene expression to promote bone remodeling and, consequently, an increased movement rate [18, 20, 21]. Recently, both preclinical and clinical studies have found that MV may enhance the OTM via a mechanism related to the induction of inflammatory mediators [14]. In addition, studies have demonstrated that AcceleDent®, a device with low-intensity vibration frequencies (30 Hz), accelerates the OTM and reduces the patient's discomfort [22].

The most notable orthodontic advance in the last decade was the introduction of digitally fabricated clear aligners to move the teeth in small and progressive sequences [23]. Currently, the largest provider of clear aligners is Invisalign® [24]. These orthodontic appliances consist of a series of plastic aligners exchanged every 7 to 14 days that deliver forces to the tooth surface, promoting a sequenced OTM [25]. It offers superior advantages regarding the comfort and hygiene of the oral cavity [26]. Moreover, different products with effects against microbial plaque that forms on clear aligners have recently reached the market, reducing the number of cavities, and consequently improving patients' gingival and periodontal health [27–29]. Clear aligners are also less uncomfortable and more aesthetic compared to conventional fixed appliances [23, 30]. This started by treating mild malocclusions only (e.g., minor crowding and space closure) but it rapidly evolved with the introduction of optimized attachments, allowing the control of more complex tooth movements [25, 28, 31, 32]. However, there are still some studies reporting problems in controlling difficult movements with clear aligners [25, 33–35]. The occurrence of unpredictable movements leads to maladjustment of the clear aligner which prolongs and compromises the treatment [36]. Therefore, the use of clear aligners to promote the OTM in complex dental problems requires more sequenced and controlled treatment.

### 1.1. Aim

The present case study aims to investigate the IL-1*β* release profile and the self-reported pain over an orthodontic treatment with clear aligners accompanied by PBM and MV. The analysis of the relationship established between these variables and the OTM complexity was also envisioned.

## 2. Case Report

The current report describes the orthodontic treatment applied to a 46-year-old female patient who presented functional and aesthetic complaints. Intraoral examination revealed a diminished overjet and overbite, rotation of the lower right second premolar (45) and upper left first premolar (24), absence of the upper left second premolar (25), an edentulous space between the lower left first premolar and the second molar (absence of the teeth 35 and 36, where it was decided to open space only for one teeth), positive buccolingual misposition of the upper left central, a tendency to a Class III malocclusion, and a deviation of the lower midline 2 mm to the left (see the X-ray imaging in the Supplementary Figure S1).

After a thorough examination of the clinical status of the patient, the Invisalign® Diamond provider and specialist in orthodontics, who supervised the orthodontic treatment (TP), decided to employ an orthodontic treatment using Invisalign® clear aligners. The evolution of the treatment was monitored through digital models acquired with the iTero Element® intraoral scanner (Copenhagen, Denmark) and intraoral photographs, before the orthodontic intervention and at each set of additional clear aligners.

Teeth 21, 24, 26, 34, 37, and 45 were selected for analysis due to their problematic and abnormal positions, and, thus, the need for more complex movements. More specifically, according to Invisalign® guidelines and classification considerations, at the beginning of this treatment, there were the following “complex/advanced” movements to perform: tooth 24 needed 55.3° of rotation, tooth 26 needed 38° of rotation, and tooth 45 needed 72° of rotation. Considering “moderate movements,” tooth 21 needed 6° of root inclination, tooth 34 needed 0.6 mm of intrusion, and tooth 37 needed 0.6 mm of extrusion. Therefore, the main goals of this orthodontic treatment were as follows: (i) in the second quadrant, a derotation with opening space between teeth 24 and 26 and root translation of teeth 11 and 21; (ii) in the third quadrant, opening space between 34 and 37); and (iii) in the fourth quadrant, a derotation of tooth number 45. The amount of tooth movement predicted by the ClinCheck® software (Align Technology, San Jose, California, USA) for each tooth is presented in Supplementary Tables 1 and S2.

Initially, only clear aligners were being used, without any kind of auxiliaries (Supplementary Figure 2). Given the complexity of the rotation movements, particularly the derotation of round teeth such as premolars and canines [19, 23–25], the clinicians decided to use orthodontic auxiliaries, namely, buttons with elastics and sectional fixed appliances, to aid the movement in the second and fourth quadrants. After a year of treatment, the first set of additional clear aligners was requested. Previous movements were maintained but with less amplitude (Supplementary Table S3). However, even after this adjustment, the clinicians noticed that the clear aligners were continually maladapted in the second and fourth quadrants (Supplementary Figure 3). For this reason, a sectional fixed appliance with an open coil-spring connecting teeth 24 and 26 was used (Supplementary Figure 4). Given the continuous misfit of the designed clear aligners, the clinicians decided to adopt orthodontic acceleration techniques—PBM and MV—to assist the desired movements.

The work described has been carried out following The Code of Ethics of the World Medical Association for experiments involving humans. The manuscript is in line with the Recommendations for the Conduct, Reporting, Editing, and Publication of Scholarly Work in Medical Journals and aims for the inclusion of representative human populations (sex, age, and ethnicity) as per those recommendations. The informed consent of the participant was obtained before experimentation, and the privacy rights of human subjects must always be observed.

### 2.1. Treatment Design and Data Acquisition

**Figure 1:**
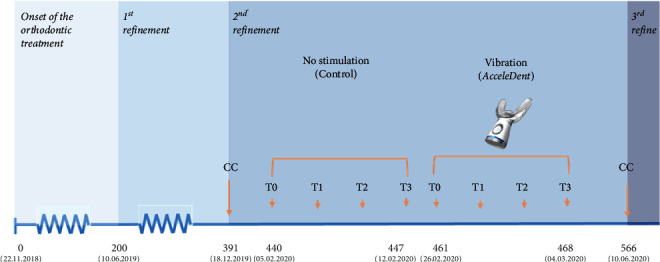
Timeline of the case report under study.= (CC : ClinCheck®; T0: baseline—before the placement of the clear aligner; T1: 24 hours after the clear aligner placement; T2: 72 hours after the clear aligner placement; T3: 7 days after the clear aligner placement).

This study comprised three stimulation phases carried out during the second (phases 1 and 2) and third (phase 3) series of additional clear aligners, since the required movements were difficult to be achieved and the continuous aligner misfit was observed. The first phase corresponds to the control moment, in which no stimulation was used. The patient was instructed to change the clear aligners every 7 days. The second phase comprised the delivery of MV, whereas the third phase included the application of PBM along with the clear aligner treatment. During phases 2 and 3, after GCF collection, the patient was instructed to change clear aligners every 5 days. The orthodontic intervention timeline is depicted in Figure 1.

The teeth associated with the most difficult movements were selected (Table 1) and three samples of GCF (per tooth) were collected to analyze the concentration of IL-1*β*. Digital records were made over time. In addition, pain monitoring was performed using a visual analogue scale (VAS) at all time points for each tooth.

The samples were collected in four moments—T0 (baseline, before the placement of the clear aligner), T1 (24 h after the clear aligner placement), T2 (72 h), and T3 (7 days), in all stimulation phases (i.e., no stimulation, MV, and PBM). After IL-1*β* collection and pain measurement at the baseline, the coil-spring between the teeth 24 and 26 was (re) activated by the length of a bracket (see Supplementary Figure S5 for further information on spring-coil specificities).

## 3. Intervention

### 3.1. Stimulation Procedures

During the second phase of the study, the patient was instructed to use the MV device AcceleDent by Propel Orthodontics® (Milpitas, California, USA) for 20 min/day. This device operates with a frequency and applied force of 30 Hz and 0.25 N, respectively [37].

In the third phase, the PBM device OrthoPulse by Biolux® Research (Vancouver, Canada) was used for 5 min per arch, totalizing 10 min/day. This stimulation device operates at a fluence of 90 mW/cm^2^ and it uses 850 nm near-infrared (NIR) light to promote bone formation around the tooth roots being dislocated [9].

### 3.2. GCF Collection and IL-1*β* Protein Quantification

The GCF was collected from the pressure side of the selected teeth. The site was isolated using cotton rolls and any supragingival plaque was removed using a cotton bud. The cervical area was dried using an air syringe, and GCF was collected using standardized sterile absorbent paper point (Roeko Paper Points 0.06 ^#^30 by Coltene/Whaledent Inc, Germany). Paper strips were inserted 1 to 2 mm into the gingival sulcus around each tooth and remained *in situ* for 30 s. Blood-contaminated strips were discarded.

After GCF collection, the strips were transferred to sterile tubes. The GCF volume was determined by weighing based on the difference in the weight of the paper before and after GCF collection and assuming a GCF density value of 1, as previously described [28]. The GCF absorbed in each paper strip was diluted in 250 *μ*L of phosphate buffer saline (PBS, pH 7.4), centrifuged (13,000*g* at 4°C for 15 min), and stored at −80°C. The GCF samples were assayed to evaluate IL-1*β* concentration using an enzyme-linked immunosorbent assay (Human IL-1*β*/IL-1F2 Quantikine HS ELISA Kit by R&D Systems, Minnesota, USA), following the manufacturer's instructions. The IL-1*β* concentration (*ρ*g/*μ*L) was calculated by dividing the amount of IL-1*β* by the volume of GCF for each sample [38].

### 3.3. Statistical Analysis

Statistical analysis was performed using the IBM SPSS® Statistics 27 (Armonk, New York, USA). First, a Shapiro–Wilk test was used to ascertain the data normality. The data did not follow a normal distribution and, thus, nonparametric tests were used. For the analysis of IL-1*β* levels and self-reported pain index throughout time, the Wilcoxon signed rank test was conducted. To compare the IL-1*β* levels and self-reported pain index between different levels of stimulation and type of movement, the Kruskal–Wallis test was used. *P* values lower than 0.05 were considered statistically significant. Graphical analysis and reporting were performed using GraphPad Prism version 6.0 for Windows (GraphPAd Software Inc, San Diego, California, USA).

## 4. Treatment Results

### 4.1. Overall Orthodontic Movement Evaluation

With the constant maladjustment, the second set of additional clear aligners was carried out and the vibration device AcceleDent® was introduced. At T0, the space in the second quadrant was open, but some essential moves were still missing (Supplementary Table S4), namely, the 1.4 mm extrusion and 3.0° mesial rotation of tooth 24, as well as a distal rotation of 26.7° of tooth 45. The clear aligner misadjusted increased over time during vibrational stimulation (Supplementary Figure S6). After 4 months of vibration, the clear aligner misfit in the second quadrant continued and intrusion of tooth 24 occurred.

Hence, the third set of additional clear aligners was necessary to induce a 0.5 mm mesial translation movement and 2.1 mm extrusion of tooth 24, and 0.1 mm distal translation movement of tooth 26. Both teeth were supposed to perform a 11° distal rotation. In the mandibula, 0.8 mm mesial translation of tooth 34, distalization movement of tooth 37, and a crown and root distal rotation of 45° of tooth 45 were envisioned (Supplementary Table S5). During this phase, OrthoPulse® was used to apply PBM. Initially, the clear aligners were fully adjusted, but, after 3 months, there was a misfit in the second and fourth quadrants. This was probably because teeth 45, 24, and 26 still require rotational movements, and tooth 24 needs to be extruded. Hence, the clear aligner showed many difficulties in gripping the tooth (Supplementary Figure S7).

**Figure 2:**
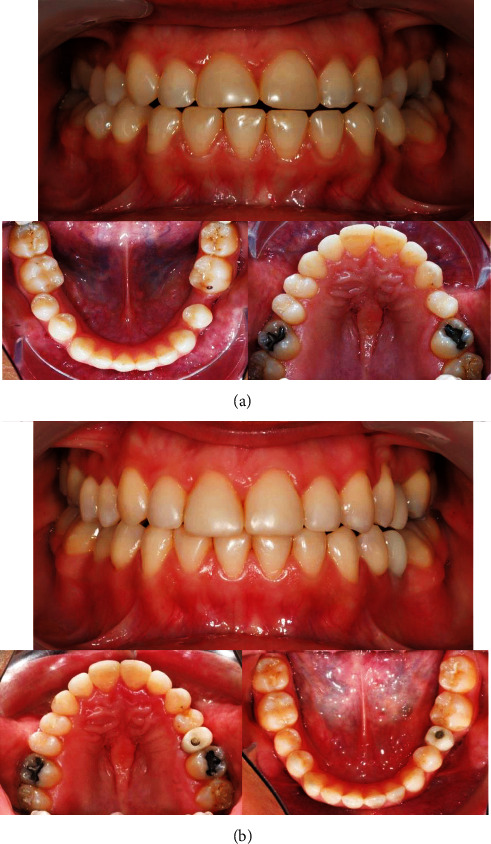
Different views of the case report before the orthodontic treatment: (a) Pretreatment photographic recording (September 20th, 2018) and (b) Posttreatment photographic recording (August 4th, 2021).

The clinical case was completed successfully (Supplementary Figure S8), although it was necessary to resort to auxiliaries in more complex movements to achieve the treatment goal (see Figure 2 with pre- and posttreatment photographic recording).

### 4.2. Longitudinal Analysis of IL-1*β* Profile and Pain

**Figure 3:**
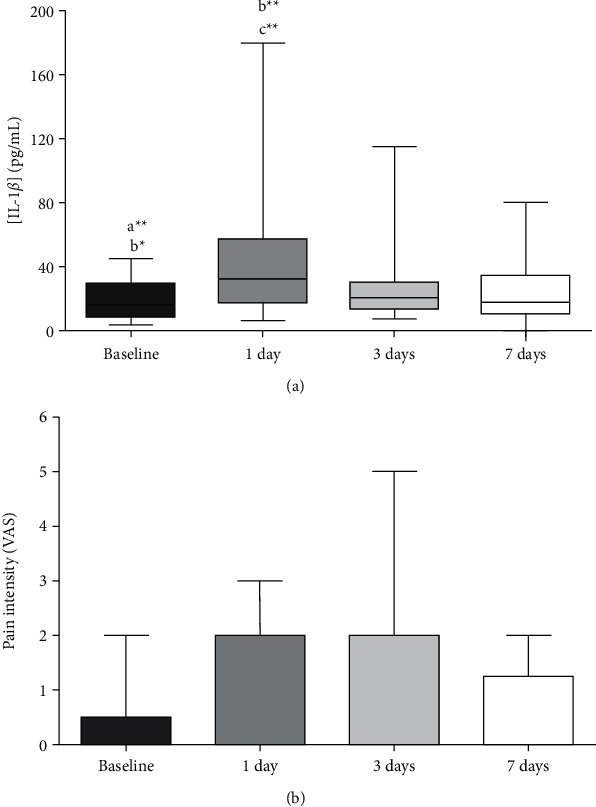
Levels of (a) interleukin-1*β* in gingival crevicular fluid samples and (b) pain intensity using visual analogue scale at baseline and after 1, 3, and 7 days of orthodontic treatment. *a* denotes significant differences compared to 1 day; *b* denotes significant differences compared to 3 days; and *c* denotes significant differences compared to 7 days (*p* < 0.05).

To evaluate the effect of time on IL-1*β* levels, independently from the type of stimulation or complexity of the orthodontic movement, the data were grouped according to the measurements' time point. The obtained graph and statistical analysis are depicted in Figure 3(a). IL-1*β* levels reached a maximum 1 day after the beginning of the study stages, which were significantly different from the levels measured at baseline and days 3 and 7. Compared to baseline, the expression of IL-1*β* was significantly increased after 1 and 3 days of activation/reactivation, demonstrating an augmented inflammatory response at these timepoints, more prominent on day 1.

A similar analysis was performed for self-reported pain using the VAS (Figure 3(b)), for which no significant differences were observed with time during the first week after the beginning/reactivation of the orthodontic treatment.

#### 4.2.1. Effect of Light and Vibration Stimulation on IL-1*β* Levels and Pain over Time

**Figure 4:**
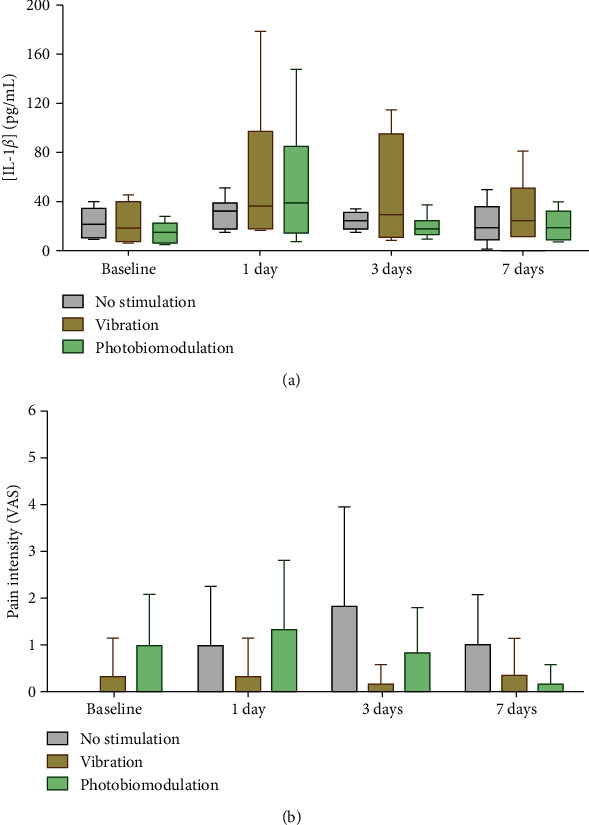
Graphical display of (a) interleukin-1*β* levels in gingival crevicular fluid samples; and (b) pain scores reported by the patient, at the baseline and after 1, 3, and 7 days, grouped according to the type of stimulation (i.e., no stimulation, vibration, and photobiomodulation).

At this point, the effect of three levels of stimulation on IL-1*β* expression and pain was assessed, independently of the type of movement induced by the OTM.  Figure 4 displays the profile of IL-1*β* and self-reported pain over time according to the type of stimulation employed. Despite there being a small tendency for an increase in the levels of IL-1*β* on day 1 after both PBM and MV, and on day 3 after MV, the great variability of the data disabled us to find a significant difference among the type of stimulation for any time point (Figure 4(a)).

In the case of the pain scores, Figure 4(b) suggests that the vibration stimulus was able to slightly reduce the self-reported pain index 1 and 3 days after stimulation when compared to the nonstimulated group. Importantly, the PBM and MV groups showed some level of pain at the baseline as it coincides with the introduction of a new set of clear aligners. Thus, the graph depicted in Figure 4(b) must be preferably analyzed by comparing the data over time within each stimulation group.

#### 4.2.2. Impact of the Type of Movement on IL-1*β* Expression and Pain over Time

**Figure 5:**
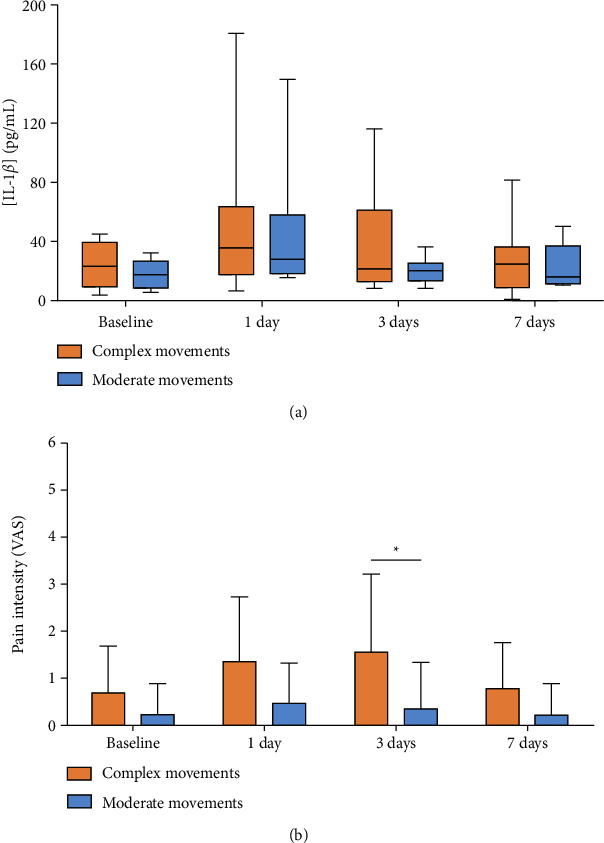
Graphical display of (a) interleukin-1*β* levels in gingival crevicular fluid samples; and (b) pain scores reported by the patient, at the baseline and after 1, 3, and 7 days, grouped according to the complexity of the movement promoted by the orthodontic appliances (i.e., complex and moderate movements) (*p* < 0.05).

We aimed to address how different types of movement and their inherent complexity could impact IL-1*β* levels and self-reported pain. For that, two groups of teeth were considered: (i) teeth with complex movements, comprising the measurement related to teeth 24, 26 (translation movement with a coil-spring), and 45 (rotation and extrusion movements), where the clear aligners were continuously maladapted; and (ii) teeth with moderate (i.e., less complex) movements, namely, teeth 21 (torque movement), 34, and 37 (translation movement without coil-spring). The corresponding data on IL-1*β* and pain are displayed over time in Figure 5. For IL-1*β* expression, similar results to those observed for different types of stimulation were found. Due to the great variability of the data (mainly for days 1 and 3), no statistically significant differences were detected between complex and moderate movements, although the graph suggests that slightly augmented levels of IL-1*β* were detected in GCF samples from teeth subjected to complex movements compared to moderate movements on day 3 (Figure 5(a)).

In line with this observation, Figure 5(b) shows a statistically significant difference between the pain felt in teeth describing complex and moderate movements on day 3. Overall, and independently from the type of stimulation, complex movements were associated with increased pain levels on the third day after orthodontic reactivation, according to the discomfort reported by the patient.

## 5. Discussion

The present case report and investigational study analyze the profile of IL-1*β* production and self-reported pain during the first week that follows the activation/reactivation of the orthodontic appliances. The orthodontic treatment started without any additional treatment, and IL-1*β* and pain data were collected at the second set of additional clear aligners (phase 1). During the third set of additional clear aligners of the orthodontic treatment, the teeth and surrounding tissues were stimulated using MV (phase 2) and PBM (phase 3), in which IL-1*β* levels were measured and pain scores were recorded. We noticed that IL-1*β* increased on the first day, regardless of the type of tooth and stimulation, and there is no relationship between increased IL-1*β* and stronger pain.

### 5.1. Inflammatory Response on Clear Aligner-Strained Teeth

Multiple reports on the analysis of GCF of patients undergoing a conventional orthodontic treatment demonstrate that several biochemical changes occur during the first weeks of treatment [3, 6, 38]. In accordance, we detected time-dependent variations in IL-1*β* levels during the first days after reactivation.

In our study, regardless of the type of tooth and stimulation, IL1-*β* was greatly increased in the 24 h that followed the beginning of the treatment. At baseline, IL-1*β* was much more reduced than on the first and third days, but there were no statistical differences compared to the seventh day, showing that it returned to baseline values. In line with these findings, Grant and colleagues and García-Lopez et al. have previously reported an increase in IL-1*β* and TNF-*α* levels from 2 to 24 h after the beginning of orthodontic treatment, returning to control levels from 24 to 48 h [22, 39]. These data confirm the activation of inflammatory mediators during the orthodontic treatment, a very early response to orthodontic stress.

In addition, we assessed how different types of movement and their inherent complexity could impact the IL-1*β* level. Grant et al. found IL-1*β* increased levels in the canines subjected to inclination using MBT prescription brackets (3 M Unitek, UK) at 4 h, 7 days, and 6 weeks, while no significant changes in IL-1*β* levels were found among time points [39]. This observation suggests that different teeth with distinct movements present different IL-1*β* expression profiles. Based on such evidence, we would expect an increase in IL-1*β* between the 24 h and 3 days follow-ups, mainly for complex movements when compared to the moderate ones. However, we found no statistically significant differences between complex and moderate movements, although the data suggest that slightly augmented levels of IL-1*β* were detected in GCF samples on day 3 (Figure 4(a)). Importantly, this inflammatory behavior was previously observed in patients using fixed orthodontic appliances [3, 38].

The misadjustment observed during the first and second sets of additional clear aligners led the clinicians to consider acceleration methods. MV have been proven to stimulate osteoclasts' differentiation from hematopoietic cells by increasing blood flow [19] and improving OTM rate via a mechanism related to the induction of inflammatory mediators [19, 40]. Similarly, PBM has been proved to stimulate the proliferation of periodontium cells to modulate the orthodontically induced inflammatory response in the periodontium due to the multiple biomechanisms, including the release of inflammatory cytokines, affecting bone remodeling and accelerating tooth movement [41–44].

Here, the effect of the three stimulation phases (i.e., no stimulation, MV, and PBM) on IL-1*β* secretion showed a small tendency for an increase on day 1 after MV and PBM, and on day 3 after MV. The great variability of the data disabled us to find significant differences between the type of stimulation for any time point, which could be caused by the distinct movements that were evaluated. Importantly, some teeth had more than one movement. In such cases, the movement with the greatest amplitude in ClinCheck® planning was considered. In addition, teeth 24, 26, and 45 required the utilization of auxiliaries, namely, brackets and a coil-spring, which could have also impacted the overall analysis.

### 5.2. Pain Indexes along the Orthodontic Treatment Using Clear Aligners

During the various phases of orthodontic treatment, pain develops in response to the tension and pressure zones generated in the periodontal ligament following the application of force [45, 46].

Here, the self-reported pain index did not change significantly with time during the first week after the beginning or reactivation of the orthodontic treatment. A statistically significant difference between the pain felt in teeth describing complex and moderate movements on day 3 was observed. Overall, and independently from the type of stimulation, complex movements tended to be associated with increased pain levels on the third day after orthodontic reactivation.

Previous studies have demonstrated that the pain reaches its peak 24 h after the beginning of the orthodontic stress, which gradually decreases over the following 7 days [47, 48]. Specifically, White et al. [44] and Bondemark et al. [49] found that pain peaked between the first and third days and gradually decreased over the fourth and fifth days. It is important to note that the current case report required the utilization of auxiliary appliances, which may have influenced the level of pain felt by the patient. This could have contributed to the different results found compared to the existing literature.

No correlation between IL-1*β* levels and pain indexes was found. In GCF, proinflammatory mediators such as IL-1*β*, prostaglandin E2 (PGE_2_), and neuropeptides (e.g., substance P) were associated with pain and discomfort [50, 51]. Giannopoulou et al. reported that pain intensity at the 1 h follow-up was associated with increased PGE_2_ levels, whereas pain intensity at 24 h was associated with increased IL-1*β* levels [50]. Sampaio et al. reported an insignificant increase in IL-1*β* levels 24 h after the onset of the force application compared to baseline [51].

MV have been suggested as a very helpful stimulation modality to simultaneously activate different nerve fibers that conduct nonnoxious stimuli, and to reduce compression of the periodontal ligament [52], which have been associated with pain relief [44, 45]. Here, we found no differences in pain scores after MV stimulation, although MV were able to slightly reduce self-reported pain index 24 h and 3 days after stimulation. This is in line with previous reports where similar levels of pain were observed in groups with and without vibration [53, 54]. On the contrary, Lobre et al. reported significantly lower pain scores during the fourth month in the group treated with AcceleDent® compared to controls [55].

Similar to what has been reported for IL-1*β* expression, the application of PBM has been effective in alleviating orthodontic pain without apparent side effects [14, 15]. PBM is thought to reduce pain by increasing local blood flow, inhibiting inflammatory substance secretion, inducing neurotransmitter release, altering the conduction and excitation of peripheral nerves, and stimulating endorphin release [14, 15]. However, data from this case report showed no significant differences in pain scores reported by the patient after PBM application compared to the other stimulation regimens. The use of different methods of pain assessment and the parameters of PBM may explain the disparity of the obtained results when compared to previous data [14, 15].

### 5.3. Effectiveness of the Idealized Orthodontic Movement

From a clinical perspective, this case report presented several clear aligner mismatches in teeth 24 and 45 due to the complex movements to be performed. Using the same clear aligner (approximately 10 months after the first collection), the vibratory stimuli were introduced in the treatment. During this phase, there was a slight misadjustment of 0.2 mm on tooth 24, with the remaining teeth being adjusted. When introducing the use of MV, it was deduced that the stimulus could somehow recover this maladjustment, but it did not happen. During the vibration phase, which lasted approximately 3 months, the tooth was misadjusted even more.

Hence, new clear aligners were made, and PBM therapy was provided over 3 months. At the end of this phase, both teeth 24 and 45 ended up misfit.

Therefore, the stimulation techniques used for movement acceleration were ineffective in this case report. Despite the long treatment time, the clear aligners always ended up misfitting on teeth that required more complex movements. Thus, we deduce that these stimulation protocols could be effective in correcting simpler clinical situations.

## 6. Conclusion and Future Perspectives

Interleukin-1*β* increased on the first day regardless of the type of tooth and stimulation. Although PBM and MV have been proven to accelerate OTM and modulate the patients' inflammatory response, the current study found no statistically significant differences between the IL-1*β* levels and pain between stimulated and nonstimulated teeth. Besides, no correlation between increased IL-1*β* and pain indexes was found. Clinically, even after stimulation, the clear aligner maladjustment remained in teeth requiring more complex movements. We found that IL-1*β* increased at the 24 h follow-up regardless of the type of tooth and stimulation, and the pain was increased on day 3. There is no correlation between increased IL-1*β* and pain indexes, but complex movements were associated with increased self-reported pain.

Overall, we observed that clear aligners show some limitations when complex tooth movements are required, even when the orthodontic treatment is combined with acceleration techniques. To counteract the considerable mismatches associated with more complex movements, cellular mechanisms of action must be well-defined before clinical application. Besides, future studies in the field must investigate optimal MV and PBM protocols (in terms of, e.g., dose, wave properties, and the application technique) to address specific types of movement and teeth. The development of customized and programmable stimulation devices designed in a way to specifically stimulate the required direction of movement could also constitute an optimized solution to achieve an effective, rapid, and painless OTM.

## Figures and Tables

**Table 1:** Description and localization of the analyzed teeth.

Teeth	**21**	**24**	**26**	**34**	**37**	**45**
Quadrant	2^nd^	2^nd^	2^nd^	3^rd^	3^rd^	4^th^
Movement type	6.0° root inclination	55.3° mesial rotation	38.0° distal rotation	0.6 mm intrusion	0.6 mm extrusion	72.0° distal rotation
Auxiliar	No	Yes (bracket + spring)	Yes (tube + spring)	No	No	No (along the collection)
Pressure side	Buccal	Mesial	Buccal	Mesial	Palatine	Distal
Tension side	Palatine	Distal	Palatine	Distal	Vestibular	Mesial

## Data Availability

No data were used to support this study.

## Supplementary Materials

Supplementary Materials Supplementary Figure S1. X-ray imaging of the case report before the orthodontic treatment obtained using the Cranex 3+ Panoramic X-ray device by Soredex Orion Corporation (Helsinki, Finland; September 20th, 2018). Supplementary Figure S2. Photographic record at the start of treatment (only wearing clear aligners, November 22nd, 2018). Supplementary Figure S3. Photographic record of the clear aligner misfit after placing sectional fixed auxiliaries to aid the translation movement before the application of photobiomodulation and vibration (November 9th, 2019). Supplementary Figure S4. Photographic record of the a) first refinement with clear aligners fully adjusted (June 19th, 2019); b) 24's intrusion with added spring (September 4th, 2019); and c) misadjusted clear aligner (October 16th, 2019). Supplementary Figure S5. Spring-coil activation of the second quadrant (teeth 24 and 26). Supplementary Figure S6. Photographic recording of the a)–c) beginning of the second refinement (January 8th, 2020) and d)–e) misfit registration (April 18th, 2020). Supplementary Figure S7. Photographic recording of the a) beginning of the third refinement (June 23rd, 2020); and b) misfit registration on teeth 24 and 45 (September 16th, 2020). Supplementary Figure S8. X-Ray imaging of the case report after the orthodontic treatment obtained using the Cranex 3+ Panoramic X-Ray device by Soredex Orion Corporation (Helsinki, Finland) (November 17th, 2021). Supplementary Table S1. Predicted movements for the maxillary arch before the onset of the orthodontic treatment. Supplementary Table S2. Predicted movements for the mandibular arch before the onset of the orthodontic treatment. Supplementary Table S3. First refinement-predicted movements for the maxillary and mandibular arches. Supplementary Table S4. Second refinement-predicted movements for the maxillary and mandibular arches. Supplementary Table S5. Third refinement-predicted movements for the maxillary and mandibular arches.
