# Corrigendum to “Human Interleukin-1*β* Profile and Self-Reported Pain Monitoring Using Clear Aligners with or without Acceleration Techniques: A Case Report and Investigational Study”

**DOI:** 10.1155/ijod/9896852

**Published:** 2025-08-30

S. Pascoal, A. Gonçalves, A. Brandão, et al., “Human Interleukin-1*β* Profile and Self-Reported Pain Monitoring Using Clear Aligners with or without Acceleration Techniques: A Case Report and Investigational Study,” *International Journal of Dentistry* 2022 (2022): 8252696, https://doi.org/10.1155/2022/8252696.

In the article, there was an error in Figure 1. The figure was cropped and the corrected Figure 1 is shown below along with the updated caption:

We apologize for this error.

## Figures and Tables

**Figure 1:**
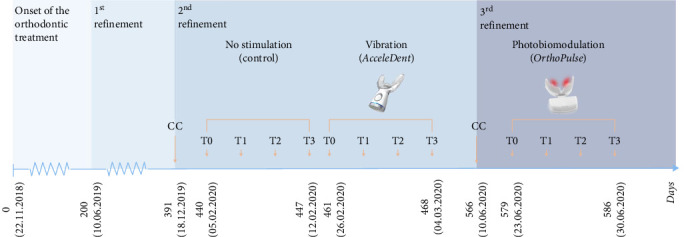
Timeline of the case report under study. (CC: ClinCheck®; T0: baseline – before the placement of the aligner; T1: 24 hours after the aligner placement; T2: 72 hours after the aligner placement; T3: 7 days after the aligner placement).

