# Supplementary material for: Human Interleukin-1β Profile and Self-Reported Pain Monitoring Using Clear Aligners with or without Acceleration Techniques: A Case Report and Investigational Study
Source: Int J Dent. 2022 Aug 31;2022:8252696. doi: 10.1155/2022/8252696 (PMC10287526; doi:10.1155/2022/8252696)
Supplement: Supplementary Materials — Supplementary Figure S1. X-ray imaging of the case report before the orthodontic treatment obtained using the Cranex 3+ Panoramic X-ray device by Soredex Orion Corporation (Helsinki, Finland; September 20th, 2018). Supplementary Figure S2. Photographic record at the start of treatment (only wearing clear aligners, November 22nd, 2018). Supplementary Figure S3. Photographic record of the clear aligner misfit after placing sectional fixed auxiliaries to aid the translation movement before the application of photobiomodulation and vibration (November 9th, 2019). Supplementary Figure S4. Photographic record of the a) first refinement with clear aligners fully adjusted (June 19th, 2019); b) 24's intrusion with added spring (September 4th, 2019); and c) misadjusted clear aligner (October 16th, 2019). Supplementary Figure S5. Spring-coil activation of the second quadrant (teeth 24 and 26). Supplementary Figure S6. Photographic recording of the a)–c) beginning of the second refinement (January 8th, 2020) and d)–e) misfit registration (April 18th, 2020). Supplementary Figure S7. Photographic recording of the a) beginning of the third refinement (June 23rd, 2020); and b) misfit registration on teeth 24 and 45 (September 16th, 2020). Supplementary Figure S8. X-Ray imaging of the case report after the orthodontic treatment obtained using the Cranex 3+ Panoramic X-Ray device by Soredex Orion Corporation (Helsinki, Finland) (November 17th, 2021). Supplementary Table S1. Predicted movements for the maxillary arch before the onset of the orthodontic treatment. Supplementary Table S2. Predicted movements for the mandibular arch before the onset of the orthodontic treatment. Supplementary Table S3. First refinement-predicted movements for the maxillary and mandibular arches. Supplementary Table S4. Second refinement-predicted movements for the maxillary and mandibular arches. Supplementary Table S5. Third refinement-predicted movements for the maxillary and mandibular arches. [file 8252696.f1.doc]

#
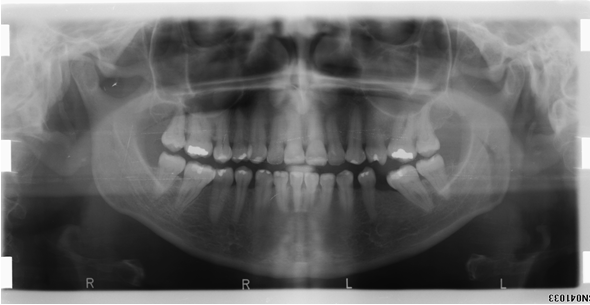
Supplementary Materials

**Supplementary Figure S1.** X-Ray imaging of the case report before the orthodontic treatment obtained using the Cranex 3+ Panoramic X-Ray device by Soredex Orion Corporation (Helsinki, Finland) (September 20^th^, 2018).


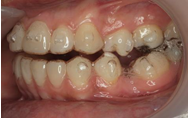


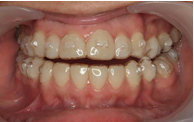

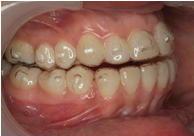


**Supplementary Figure S2.** Photographic record at the start of treatment, only wearing clear aligners (November 22^nd^, 2018).


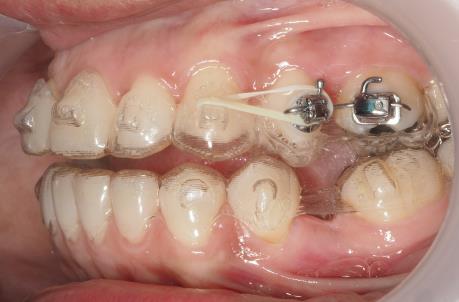

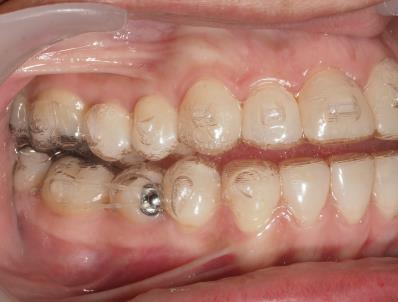


**Supplementary Figure S3.** Photographic record of the aligner misfit after placing sectional fixed auxiliaries to aid the translation movement before the application of photobiomodulation and vibration (November 9^th^, 2019).


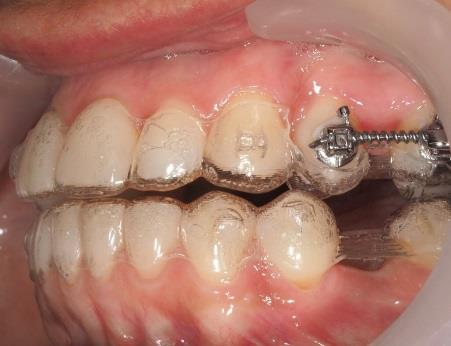

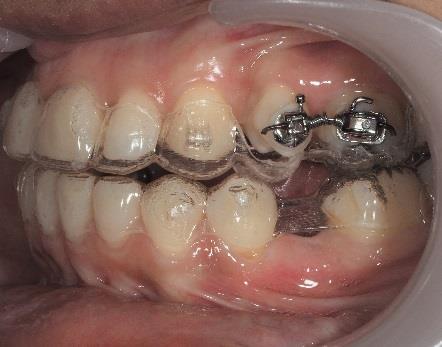

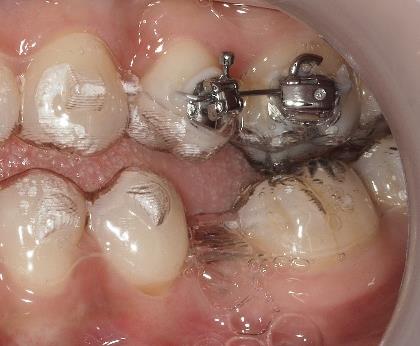


**a)**

**b)**

**c)**

**Supplementary Figure S4.** Photographic record of the **a)** first refinement with clear aligners fully adjusted (June 19^th^, 2019); **b)** 24’s intrusion with added spring (September 4^th^, 2019); **c)** misadjusted aligner (October 16^th^, 2019).


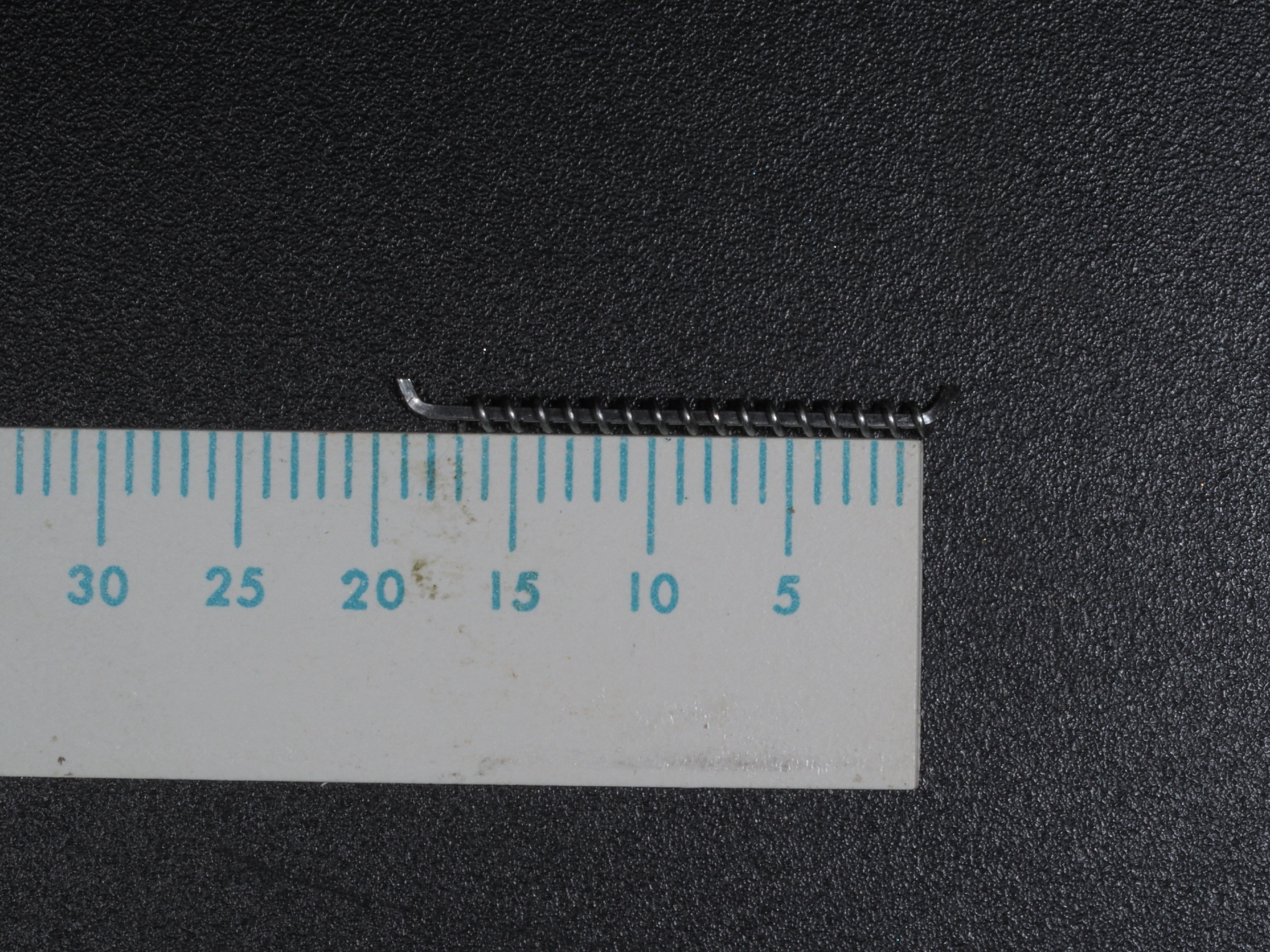

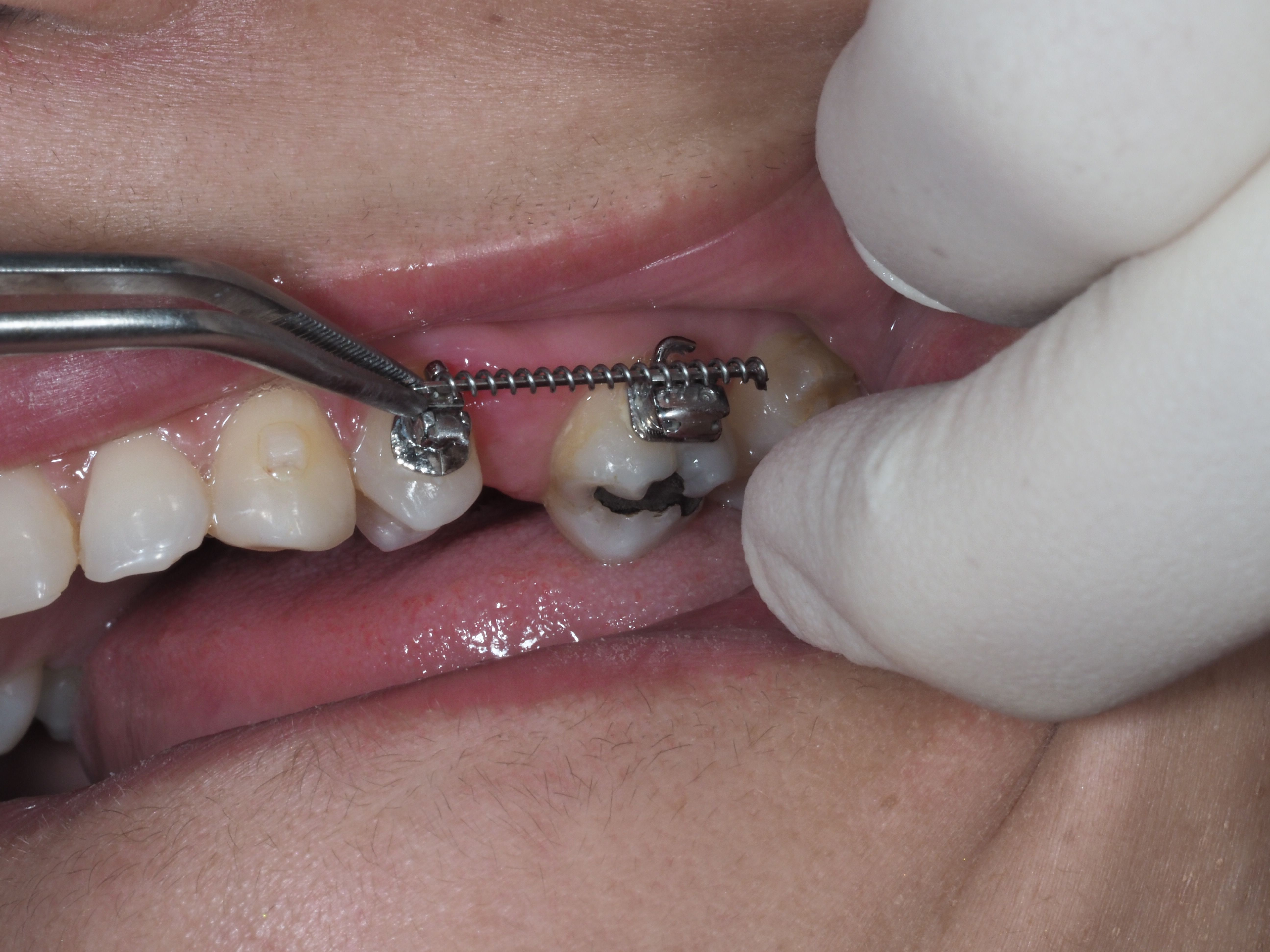

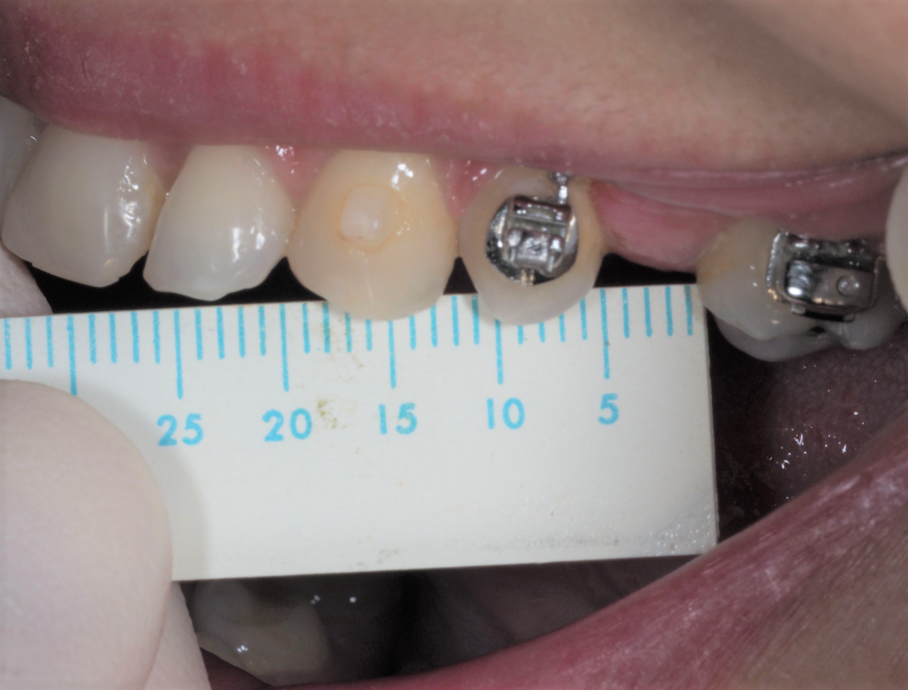


**Supplementary Figure S5.** Spring-coil activation of the second quadrant (teeth 24 and 26).


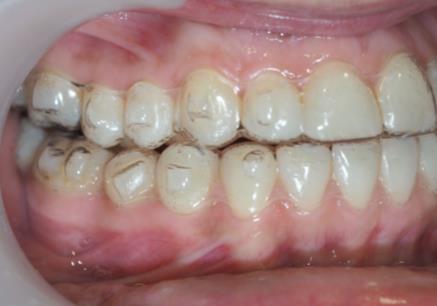

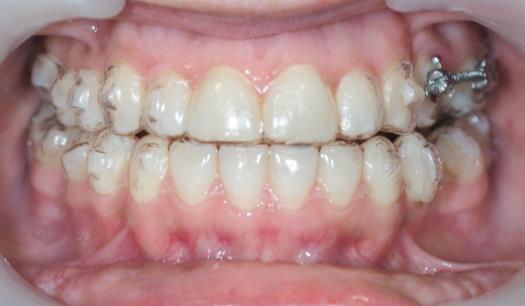

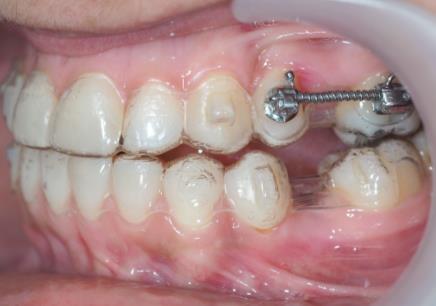

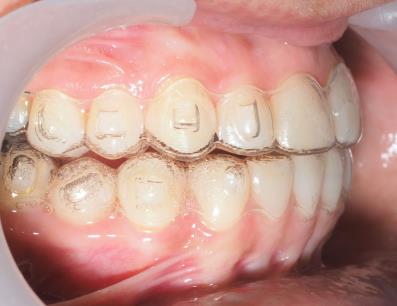

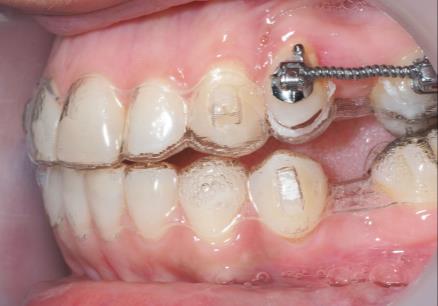


**a)**

**b)**

**c)**

**d)**

**e)**

**Supplementary Figure S6.** Photographic recording of the **a)-c)** beginning of the second refinement (January 8^th^, 2020); and **d)-e)** misfit registration (April 18^th^, 2020).


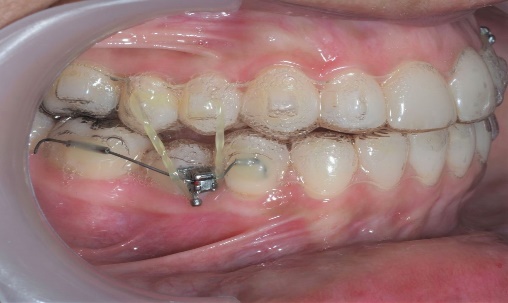

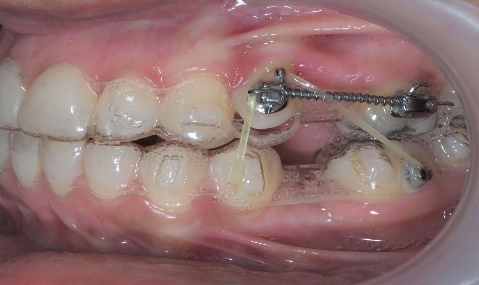


**a)**

**b)**

**Supplementary Figure S7.** Photographic recording of the **a)** beginning of the third refinement (June 23^rd^, 2020); and **b)** misfit registration on teeth 24 and 45 (September 16^th^, 2020).


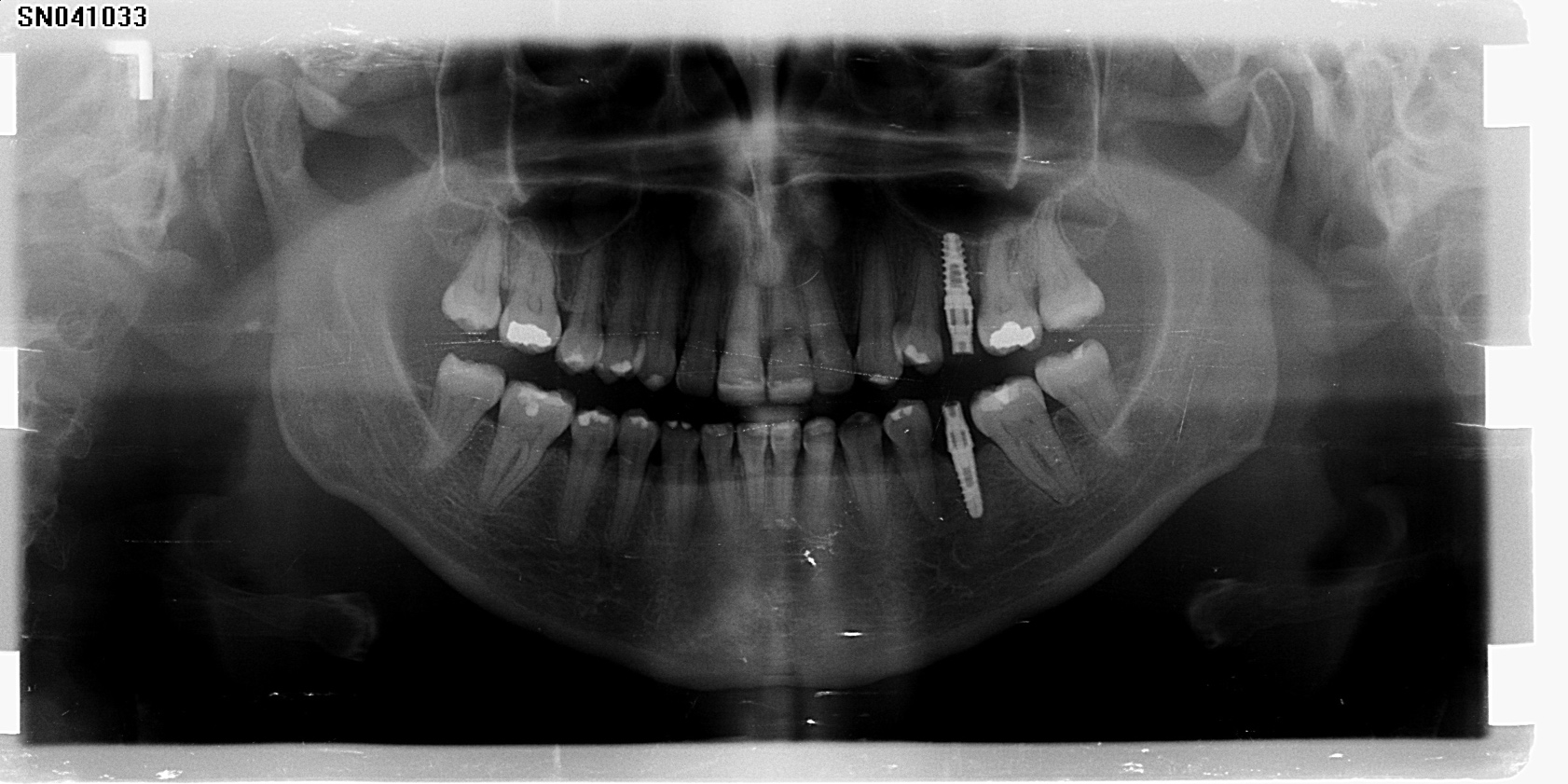


**Supplementary Figure S8.** X-Ray imaging of the case report after the orthodontic treatment obtained using the Cranex 3+ Panoramic X-Ray device by Soredex Orion Corporation (Helsinki, Finland) (November 17^th^, 2021).

**Supplementary Table S1. Predicted movements for the maxillary arch before the onset of the orthodontic treatment.**

| **Upper Crown** | 18 | 17 | 16 | 15 | 14 | 13 | 12 | 11 | 21 | 22 | 23 | 24 | 25 | 26 | 27 | 28 |
| --- | --- | --- | --- | --- | --- | --- | --- | --- | --- | --- | --- | --- | --- | --- | --- | --- |
| Extrusion/Intrusion, mm | - | 0.1 E | 0 | 0.5 I | 0 | 0.9 E | 0.3 E | 0.5 E | 0.7 E | 1.1 E | 1.9 E | 0 | - | - | 0.4 E | 0 |
| Relative Ext/Int, mm | - | 0.9 E | 0.1 I | 0.1 E | 0.3 E | 0.6 E | 0.3 E | 1.2 E | 1.1 E | 0.5 E | 0.6 E | 0.5 I | - | - | 0.3 E | 0.3 E |
| Translation Buccal/Lingual | - | 0.8 L | 1.1 B | 1.0 B | 0.9 B | 0.6 B | 0.1 B | 0.6 L | 0.2 L | 0.9 B | 1.8 B | 2.6 B | - | - | 2.6 B | 0.5 B |
| Translation Mesial/Distal | - | 0.1 D | 0.8 M | 0.2 D | 1.0 D | 1.2 D | 0.2 D | 0.1 D | 0.2 D | 0.3 D | 0.4 D | 1.7 M | - | - | 1.1 D | 2.1 D |
| Rotation, ° | - | 5.6 D | 27.2 D | 24.3 D | 6.0 M | 9.0 M | 10.9 M | 5.6 M | 7.9 M | 3.0 M | 3.7 D | 55.3 M | - | - | 38.0 D | 8.3 M |
| Angulation, ° | - | 3.2 D | 1.5 D | 7.8 D | 7.4 D | 5.0 D | 1.5 M | 0.2 D | 3.5 M | 6.8 M | 3.7 M | 0 | - | - | 13.8 D | 1.1 D |
| Inclination, ° | - | 9.0 L | 2.4 L | 9.1 L | 6.5 L | 4.8 L | 4.3 L | 11.6 L | 6.0 L | 4.3 L | 0.8 L | 0.5 L | - | - | 6.5 L | 10.2 L |

| **Upper Root** | 18 | 17 | 16 | 15 | 14 | 13 | 12 | 11 | 21 | 22 | 23 | 24 | 25 | 26 | 27 | 28 |
| --- | --- | --- | --- | --- | --- | --- | --- | --- | --- | --- | --- | --- | --- | --- | --- | --- |
| Extrusion/Intrusion, mm | - | 0.1 E | 0 | 0.5 I | 0 | 0.9 E | 0.3 E | 0.5 E | 0.7 E | 1.1 E | 1.9 E | 0 | - | - | 0.4 E | 0 |
| Relative Ext/Int, mm | - | - | - | - | - | - | - | - | - | - | - | - | - | - | - | - |
| Translation Buccal/Lingual | - | 1.8 B | 1.8 B | 3.9 B | 2.8 B | 2.5 B | 1.5 B | 3.2 B | 2.3 B | 2.3 B | 2.1 B | 2.7 B | - | - | 4.6 B | 3.5 B |
| Translation Mesial/Distal | - | 0.8 M | 1.2 M | 2.2 M | 1.2 M | 0.8 M | 0.7 D | 0 | 1.4 D | 2.5 D | 1.9 D | 1.7 M | - | - | 3.0 M | 1.8 D |
| Rotation, ° | - | 5.6 D | 27.2 D | 24.3 D | 6.0 M | 9.0 M | 10.9 M | 5.6 M | 7.9 M | 3.0 M | 3.7 D | 55.3 M | - | - | 38.0 D | 8.3 M |
| Angulation, ° | - | 3.2 M | 1.5 M | 7.8 M | 7.4 M | 5.0 M | 1.5 D | 0.2 M | 3.5 D | 6.8 D | 3.7 D | 0 | - | - | 13.8 M | 1.1 M |
| Inclination, ° | - | 9.0 B | 2.4 B | 9.1 B | 6.5 B | 4.8 B | 4.3 B | 11.6 B | 6.0 B | 4.3 B | 0.8 B | 0.5 B | - | - | 6.5 B | 10.2 B |

**Supplementary Table S2.** Predicted movements for the mandibular arch before the onset of the orthodontic treatment.

| **Lower Crown** | 48 | 47 | 46 | 45 | 44 | 43 | 42 | 41 | 31 | 32 | 33 | 34 | 35 | 36 | 37 | 38 |
| --- | --- | --- | --- | --- | --- | --- | --- | --- | --- | --- | --- | --- | --- | --- | --- | --- |
| Extrusion/Intrusion, mm | - | 0 | 0.2 E | 0.1 E | 0.1 E | 0.2 E | 0.8 I | 0.8 I | 0.5 I | 0.3 I | 0.4 I | 0.6 I | - | - | 0.6E | 0 |
| Relative Ext/Int, mm | - | 0.1 E | 0.2 E | 0.6 E | 0.1 I | 0.4 E | 0.4 I | 0.3 I | 0.1 I | 0 | 0.4 I | 0.7 I | - | - | 1.1 E | 0.4 E |
| Translation Buccal/Lingual | - | 0.4 L | 0.4 B | 2.5 L | 0.4 L | 0.6 L | 0.9 L | 1.1 L | 1.1 L | 0.8 L | 0.1 B | 0.1 B | - | - | 0.6 B | 0.3 B |
| Translation Mesial/Distal | - | 0 | 0.2 M | 0.3 D | 0.2 D | 0.9 D | 0.4 D | 0 | 0.2 D | 0.6 D | 0.8 D | 0.2 M | - | - | 1.6 D | 1.6 D |
| Rotation, ° | - | 0.2 M | 4.4 D | 72.0 D | 8.3 D | 11.1 M | 1.9 D | 3.0 M | 2.8 M | 3.9 M | 5.8 M | 19.5 M | - | - | 1.4 D | 0.9 D |
| Angulation, ° | - | 0.6 M | 5.2 D | 5.3 M | 4.7 M | 5.8 M | 2.4 D | 0.6 D | 0.1 D | 1.0 D | 4.3 M | 4.2 D | - | - | 13.4 D | 1.4 M |
| Inclination, ° | - | 2.1 L | 3.7 B | 19.0 L | 4.8 B | 0.9 B | 0.1 L | 0.1 B | 1.3 B | 1.3 L | 0.9 B | 5.0 B | - | - | 1.9 B | 1.2 B |

| **Lower Root** | 48 | 47 | 46 | 45 | 44 | 43 | 42 | 41 | 31 | 32 | 33 | 34 | 35 | 36 | 37 | 38 |
| --- | --- | --- | --- | --- | --- | --- | --- | --- | --- | --- | --- | --- | --- | --- | --- | --- |
| Extrusion/Intrusion, mm | - | 0 | 0.2 E | 0.1 E | 0.1 E | 0.2 E | 0.8 I | 0.8 I | 0.5 I | 0.3 I | 0.4 I | 0.6 I | - | - | 0.6E | 0 |
| Relative Ext/Int, mm | - | - | - | - | - | - | - | - | - | - | - | - | - | - | - | - |
| Translation Buccal/Lingual | - | 0.2 B | 0.7 L | 3.9 B | 1.8 L | 0.9 L | 0.9 L | 1.1 L | 1.5 L | 0.4 L | 0.3 L | 1.4 L | - | - | 0.1 B | 0.1 L |
| Translation Mesial/Distal | - | 0.2 D | 1.7 M | 2.0 D | 1.6 D | 3.1 D | 0.4 M | 0.2 M | 0.2 D | 0.3 D | 2.5 D | 1.5 M | - | - | 2.2 M | 2.0 D |
| Rotation, ° | - | 0.2 M | 4.4 D | 72.0 D | 8.3 D | 11.1 M | 1.9 D | 3.0 M | 2.8 M | 3.9 M | 5.8 M | 19.5 M | - | - | 1.4 D | 0.9 D |
| Angulation, ° | - | 0.6 D | 5.2 M | 5.3 D | 4.7 D | 5.8 D | 2.4 M | 0.6 M | 0.1 M | 1.0 M | 4.3 D | 4.2 M | - | - | 13.4 M | 1.4 D |
| Inclination, ° | - | 2.1 B | 3.7 L | 19.0 B | 4.8 L | 0.9 L | 0.1 B | 0.1 L | 1.3 L | 1.3 B | 0.9 L | 5.0 L | - | - | 1.9 L | 1.2 L |

**Supplementary Table S3.** First refinement-predicted movements for the maxillary and mandibular arches.

| **Upper Crown** | 18 | 17 | 16 | 15 | 14 | 13 | 12 | 11 | 21 | 22 | 23 | 24 | 25 | 26 | 27 | 28 |
| --- | --- | --- | --- | --- | --- | --- | --- | --- | --- | --- | --- | --- | --- | --- | --- | --- |
| Extrusion/Intrusion, mm | - | 0 | 0.8 E | 0.2 I | 0 | 0.9 E | 0.1 E | 0 | 0 | 0.1 E | 1.7 E | 0.9 E | - | - | 0.6 E | 0.1 E |
| Relative Ext/Int, mm | - | 0.4 E | 1.3 E | 0.7 E | 0.3 E | 0.7 E | 0.1 E | 0.6 E | 0.3 E | 0.1 I | 1.0 E | 0.8 E | - | - | 1.5 E | 0.5 E |
| Translation Buccal/Lingual | - | 0.2 L | 0 | 0.3 B | 0.7 B | 0.4 B | 0.1 L | 0.8 L | 0.4 L | 0.4 B | 0.9 B | 1.1 B | - | - | 0.8 B | 0.5 B |
| Translation Mesial/Distal | - | 0.1 M | 0.2 M | 0 | 0.2 D | 0.5 D | 0.3 D | 0.1 M | 0.2 D | 0.3 D | 0.1 M | 1.5 M | - | - | 1.5 D | 0.9 D |
| Rotation, ° | - | 1.8 D | 16.3 D | 12.0 D | 1.1 M | 11.3 M | 10.4 M | 4.0 M | 4.0 M | 6.5 M | 1.6 D | 25.5 M | - | - | 34.0 D | 0.3 M |
| Angulation, ° | - | 2.6 D | 6.0 D | 2.8 D | 2.6 D | 1.2 D | 9.5 M | 0.4 D | 1.9 D | 7.8 M | 2.1 M | 3.1 D | - | - | 11.4 D | 4.3 M |
| Inclination, ° | - | 3.7 L | 7.0 L | 13.6 L | 9.0 L | 3.5 L | 1.5 L | 4.1 L | 3.0 L | 0.9 L | 2.5 L | 6.3 L | - | - | 11.0 L | 9.7 L |

| **Upper Root** | 18 | 17 | 16 | 15 | 14 | 13 | 12 | 11 | 21 | 22 | 23 | 24 | 25 | 26 | 27 | 28 |
| --- | --- | --- | --- | --- | --- | --- | --- | --- | --- | --- | --- | --- | --- | --- | --- | --- |
| Extrusion/Intrusion, mm | - | 0 | 0.8 E | 0.2 I | 0 | 0.9 E | 0.1 E | 0 | 0 | 0.1 E | 1.7 E | 0.9 E | - | - | 0.6 E | 0.1 E |
| Relative Ext/Int, mm | - | - | - | - | - | - | - | - | - | - | - | - | - | - | - | - |
| Translation Buccal/Lingual | - | 0.9 B | 2.1 B | 4.5 B | 3.3 B | 1.9 B | 0.4 B | 0.6 B | 0.6 B | 0.7 B | 1.9 B | 3.2 B | - | - | 4.2 B | 3.3 B |
| Translation Mesial/Distal | - | 0.8 M | 2.0 M | 0.9 M | 0.5 M | 0 | 3.4 D | 0.2 M | 0.4 M | 2.9 D | 0.7 D | 2.6 M | - | - | 1.9 M | 2.1 D |
| Rotation, ° | - | 1.8 D | 16.3 D | 12.0 D | 1.1 M | 11.3 M | 10.4 M | 4.0 M | 4.0 M | 6.5 M | 1.6 D | 25.5 M | - | - | 34.0 D | 0.3 M |
| Angulation, ° | - | 2.6 M | 6.0 M | 2.8 M | 2.6 M | 1.2 M | 9.5 D | 0.4 M | 1.9 M | 7.8 D | 2.1 D | 3.1 M | - | - | 11.4 M | 4.3 D |
| Inclination, ° | - | 3.7 B | 7.0 B | 13.6 B | 9.0 B | 3.5 B | 1.5 B | 4.1 B | 3.0 B | 0.9 B | 2.5 B | 6.3 B | - | - | 11.0 B | 9.7 B |

| **Lower Crown** | 48 | 4. | 46 | 45 | 44 | 43 | 42 | 41 | 31 | 32 | 33 | 34 | 35 | 36 | 37 | 38 |
| --- | --- | --- | --- | --- | --- | --- | --- | --- | --- | --- | --- | --- | --- | --- | --- | --- |
| Extrusion/Intrusion, mm | - | 0.4 E | 0.5 E | 0.7 E | 0 | 0.1 E | 0.5 I | 0.7 I | 0.5 I | 0.2 I | 0.1 I | 0.3 E | - | - | 0.5 E | 0.5 E |
| Relative Ext/Int, mm | - | 0.7 E | 0.8 E | 1.0 E | 0 | 0.4 E | 0.1 I | 0.3 I | 0.2 I | 0.1 E | 0 | 0.3 E | - | - | 0.8 E | 0.5 E |
| Translation Buccal/Lingual | - | 0.6 L | 0.1 L | 1.6 L | 0.6 L | 0.7 L | 0.9 L | 0.9 L | 1.0 L | 0.7 L | 0.3 L | 0.1 L | - | - | 0.1 L | 0.3 L |
| Translation Mesial/Distal | - | 0.7 D | 0.5 D | 0.8 D | 0.5 D | 0.9 D | 0.5 D | 0.1 D | 0.1 D | 0.5 D | 0.6 D | 0.1 D | - | - | 0.9 D | 0.7 D |
| Rotation, ° | - | 0.6 D | 12.1 D | 50.3 D | 5.5 D | 4.1 M | 3.0 D | 1.5 M | 1.1 M | 0.8 M | 2.6 M | 8.2 M | - | - | 9.0 D | 0.2 M |
| Angulation, ° | - | 0.9 D | 7.0 D | 5.9 M | 1.7 M | 7.5 M | 0.9 D | 0.5 M | 0 | 0.3 M | 5.9 M | 7.3 D | - | - | 7.8 D | 3.6 M |
| Inclination, ° | - | 4.6 L | 0.7 L | 8.0 L | 0.1 B | 0 | 0.2 L | 1.5 B | 2.7 B | 0.5 B | 1.7 B | 1.6 B | - | - | 2.0 B | 0.2 B |

| **Lower Root** | 48 | 47 | 46 | 45 | 44 | 43 | 42 | 41 | 31 | 32 | 33 | 34 | 35 | 36 | 37 | 38 |
| --- | --- | --- | --- | --- | --- | --- | --- | --- | --- | --- | --- | --- | --- | --- | --- | --- |
| Extrusion/Intrusion, mm | - | 0.4 E | 0.5 E | 0.7 E | 0 | 0.1 E | 0.5 I | 0.7 I | 0.5 I | 0.2 I | 0.1 I | 0.3 E | - | - | 0.5 E | 0.5 E |
| Relative Ext/Int, mm | - | - | - | - | - | - | - | - | - | - | - | - | - | - | - | - |
| Translation Buccal/Lingual | - | 0.7 B | 0.1 B | 1.2 B | 0.7 L | 0.7 L | 0.8 L | 1.4 L | 1.8 L | 0.9 L | 0.9 L | 0.5 L | - | - | 0.7 L | 0.4 L |
| Translation Mesial/Distal | - | 0.4 D | 1.6 M | 2.7 D | 1.1 D | 3.9 D | 0.2 D | 0.3 D | 0.1 D | 0.6 D | 2.9 D | 2.1 M | - | - | 1.5 M | 1.8 D |
| Rotation, ° | - | 0.6 D | 12.1 D | 50.3 D | 5.5 D | 4.1 M | 3.0 D | 1.5 M | 1.1 M | 0.8 M | 2.6 M | 8.2 M | - | - | 9.0 D | 0.2 M |
| Angulation, ° | - | 0.9 M | 7.0 M | 5.9 D | 1.7 D | 7.5 D | 0.9 M | 0.5 D | 0 | 0.3 D | 5.9 D | 7.3 M | - | - | 7.8 M | 3.6 D |
| Inclination, ° | - | 4.6 B | 0.7 B | 8.0 B | 0.1 L | 0 | 0.2 B | 1.5 L | 2.7 L | 0.5 L | 1.7 L | 1.6 L | - | - | 2.0 L | 0.2 L |

**Supplementary Table S4**. Second refinement-predicted movements for the maxillary and mandibular arches.

| **Upper Crown** | 18 | 17 | 16 | 15 | 14 | 13 | 12 | 11 | 21 | 22 | 23 | 24 | 25 | 26 | 27 | 28 |
| --- | --- | --- | --- | --- | --- | --- | --- | --- | --- | --- | --- | --- | --- | --- | --- | --- |
| Extrusion/Intrusion, mm | - | 0.1 I | 0.2 E | 0.3 I | 0 | 1.0 E | 0.4 E | 0 | 0.1 I | 0.2 E | 1.8 E | 1.4 E | - | - | 0.2 E | 0.3 E |
| Relative Ext/Int, mm | - | 0.8 E | 0.6 E | 0.4 E | 0.5 E | 0.5 E | 0.1 E | 0 | 0.1 I | 0.2 I | 0.7 E | 0.7 E | - | - | 1.1 E | 1.1 E |
| Translation Buccal/Lingual | - | 0 | 0 | 0.3 B | 0.5 B | 0.8 B | 0.5 B | 0.3 B | 0.4 B | 0.8 B | 1.3 B | 1.4 B | - | - | 0 | 0.4 L |
| Translation Mesial/Distal | - | 0.3 M | 0.2 M | 0.2 M | 0.1 M | 0.4 D | 0.4 D | 0.4 D | 0.4 M | 0.5 M | 1.1 M | 1.1 M | - | - | 0.1 D | 0 |
| Rotation, ° | - | 0.5 D | 7.0 D | 7.8 D | 1.0 M | 8.1 M | 6.2 M | 0.5 M | 6.3 M | 3.5 M | 1.3 D | 3.0 M | - | - | 17.2 D | 1.6 M |
| Angulation, ° | - | 4.4 D | 3.8 D | 6.3 D | 1.5 D | 1.6 D | 10.1 M | 1.6 M | 0 | 9.3 M | 0.8 M | 2.9 D | - | - | 3.8 D | 5.1 M |
| Inclination, ° | - | 12.6 L | 6.0 L | 10.5 L | 10.7 L | 2.3 L | 2.0 L | 3.8 L | 3.9 L | 3.5 L | 2.5 L | 0.5 L | - | - | 8.2 L | 12.5 L |

| **Upper Root** | 18 | 17 | 16 | 15 | 14 | 13 | 12 | 11 | 21 | 22 | 23 | 24 | 25 | 26 | 27 | 28 |
| --- | --- | --- | --- | --- | --- | --- | --- | --- | --- | --- | --- | --- | --- | --- | --- | --- |
| Extrusion/Intrusion, mm | - | 0.1 I | 0.2 E | 0.3 I | 0 | 1.0 E | 0.4 E | 0 | 0.1 I | 0.2 E | 1.8 E | 1.4 E | - | - | 0.2 E | 0.3 E |
| Relative Ext/Int, mm | - | - | - | - | - | - | - | - | - | - | - | - | - | - | - | - |
| Translation Buccal/Lingual | - | 3.7 B | 1.9 B | 3.6 B | 3.7 B | 1.7 B | 1.1 B | 1.6 B | 1.7 B | 2.0 B | 2.3 B | 1.6 B | - | - | 2.5 B | 3.5 B |
| Translation Mesial/Distal | - | 1.6 M | 1.5 M | 2.2 M | 0.5 M | 0.2 M | 3.7 D | 0.9 D | 0.4 M | 2.5 D | 0.7 M | 1.9 M | - | - | 1.0 M | 1.6 D |
| Rotation, ° | - | 0.5 D | 7.0 D | 7.8 D | 1.0 M | 8.1 M | 6.2 M | 0.5 M | 6.3 M | 3.5 M | 1.3 D | 3.0 M | - | - | 17.2 D | 1.6 M |
| Angulation, ° | - | 4.4 M | 3.8 M | 6.3 M | 1.5 M | 1.6 M | 10.1 D | 1.6 D | 0 | 9.3 D | 0.8 D | 2.9 M | - | - | 3.8 M | 5.1 D |
| Inclination, ° | - | 12.6 B | 6.0 B | 10.5 B | 10.7 B | 2.3 B | 2.0 B | 3.8 B | 3.9 B | 3.5 B | 2.5 B | 0.5 B | - | - | 8.2 B | 12.5 B |

| **Lower Crown** | 48 | 47 | 46 | 45 | 44 | 43 | 42 | 41 | 31 | 32 | 33 | 34 | 35 | 36 | 37 | 38 |
| --- | --- | --- | --- | --- | --- | --- | --- | --- | --- | --- | --- | --- | --- | --- | --- | --- |
| Extrusion/Intrusion, mm | - | 0.1 I | 0.2 E | 0.6 E | 0.1 E | 0.1 I | 0.6 I | 0.7 I | 0.7 I | 0.1 I | 0.4 E | 0.5 E | - | - | 0.3 E | 0.2 E |
| Relative Ext/Int, mm | - | 0.2 E | 0.2 E | 0.7 E | 0 | 0.2 I | 0.7 I | 0.8 I | 0.7 I | 0.2 I | 0.1 E | 0.5 E | - | - | 0.3 E | 0.2 E |
| Translation Buccal/Lingual | - | 1.2 L | 0.7 L | 0.9 L | 0.9 L | 0.4 B | 0.2 B | 0 | 0 | 0.2 B | 0.5 B | 0.1 L | - | - | 0.2 L | 0.3 L |
| Translation Mesial/Distal | - | 0.6 D | 0.5 D | 0.4 D | 0.4 D | 0.1 D | 0 | 0 | 0.1 D | 0.2 D | 0.1 D | 0.3 D | - | - | 0 | 0 |
| Rotation, ° | - | 0.2 D | 4.0 D | 26.7 D | 14.2 D | 7.1 M | 1.6 M | 0.9 M | 2.7 M | 4.3 M | 6.2 M | 7.3 M | - | - | 4.6 D | 1.2 D |
| Angulation, ° | - | 0.8 D | 5.4 D | 1.3 M | 5.6 M | 10.4 M | 0.3 D | 0.9 M | 0.3 M | 4.2 M | 8.8 M | 4.2 D | - | - | 2.5 D | 0.1 D |
| Inclination, ° | - | 6.2 L | 0.1 B | 2.1 L | 2.1 B | 3.7 B | 1.9 B | 4.0 B | 4.1 B | 1.3 B | 2.6 B | 0.5 B | - | - | 1.7 B | 1.1 L |

| **Lower Root** | 48 | 47 | 46 | 45 | 44 | 43 | 42 | 41 | 31 | 32 | 33 | 34 | 35 | 36 | 37 | 38 |
| --- | --- | --- | --- | --- | --- | --- | --- | --- | --- | --- | --- | --- | --- | --- | --- | --- |
| Extrusion/Intrusion, mm | - | 0.1 I | 0.2 E | 0.6 E | 0.1 E | 0.1 I | 0.6 I | 0.7 I | 0.7 I | 0.1 I | 0.4 E | 0.5 E | - | - | 0.3 E | 0.2 E |
| Relative Ext/Int, mm | - | - | - | - | - | - | - | - | - | - | - | - | - | - | - | - |
| Translation Buccal/Lingual | - | 0.6 B | 0.8 L | 0.2 L | 1.5 L | 1.1 L | 0.4 L | 1.2 L | 1.3 L | 0.2 L | 0.5 L | 0.3 L | - | - | 0.7 L | 0 |
| Translation Mesial/Distal | - | 0.4 D | 1.2 M | 0.9 D | 2.1 D | 4.2 D | 0.1 M | 0.3 D | 0.2 D | 1.5 D | 3.5 D | 1.0 M | - | - | 0.8 M | 0 |
| Rotation, ° | - | 0.2 D | 4.0 D | 26.7 D | 14.2 D | 7.1 M | 1.6 M | 0.9 M | 2.7 M | 4.3 M | 6.2 M | 7.3 M | - | - | 4.6 D | 1.2 D |
| Angulation, ° | - | 0.8 M | 5.4 M | 1.3 D | 5.6 D | 10.4 D | 0.3 M | 0.9 D | 0.3 D | 4.2 D | 8.8 D | 4.2 M | - | - | 2.5 M | 0.1 M |
| Inclination, ° | - | 6.2 B | 0.1 L | 2.1 B | 2.1 L | 3.7 L | 1.9 L | 4.0 L | 4.1 L | 1.3 L | 2.6 L | 0.5 L | - | - | 1.7 L | 1.1 B |

**Supplementary Table S5**. Third refinement-predicted movements for the maxillary and mandibular arches.

| **Upper Crown** | 18 | 17 | 16 | 15 | 14 | 13 | 12 | 11 | 21 | 22 | 23 | 24 | 25 | 26 | 27 | 28 |
| --- | --- | --- | --- | --- | --- | --- | --- | --- | --- | --- | --- | --- | --- | --- | --- | --- |
| Extrusion/Intrusion, mm | - | 0.1 E | 0.6 E | 0.3 E | 0.3 I | 0.6 E | 0.3 E | 0.1 E | 0 | 0.1 E | 1.6 E | 2.1 E | - | - | 0.4 E | 0.4 E |
| Relative Ext/Int, mm | - | 0.7 E | 1.1 E | 0.6 E | 0.1 I | 0.3 E | 0 | 0.1 E | 0.1 E | 0.1 I | 0.7 E | 2.0 E | - | - | 1.6 E | 1.4 E |
| Translation Buccal/Lingual | - | 0 | 0.2 B | 0.3 B | 0.2 B | 0.4 B | 0.4 B | 0 | 0.1 B | 0.5 B | 0.8 B | 0.5 B | - | - | 0 | 0 |
| Translation Mesial/Distal | - | 0.2 M | 0.2 M | 0 | 0.1 M | 0.2 D | 0.3 D | 0.1 D | 0.1 M | 0 | 0.5 M | 0.5 M | - | - | 0.1 D | 0.1 D |
| Rotation, ° | - | 1.0 M | 5.5 D | 1.5 D | 2.5 D | 0 | 6.8 M | 1.3 M | 3.9 M | 0.7 M | 0 | 11.0 D | - | - | 11.0 D | 0.3 M |
| Angulation, ° | - | 3.1 D | 5.6 D | 5.0 D | 2.4 D | 2.7 D | 11.0 M | 2.2 M | 0.8 D | 8.2 M | 1.4 D | 6.6 D | - | - | 3.9 D | 4.2 M |
| Inclination, ° | - | 6.4 L | 5.6 L | 5.2 L | 3.6 L | 1.0 B | 0.6 B | 1.1 L | 2.0 L | 2.4 L | 0.4 B | 0.9 B | - | - | 10.5 L | 11.4 L |

| **Upper Root** | 18 | 17 | 16 | 15 | 14 | 13 | 12 | 11 | 21 | 22 | 23 | 24 | 25 | 26 | 27 | 28 |
| --- | --- | --- | --- | --- | --- | --- | --- | --- | --- | --- | --- | --- | --- | --- | --- | --- |
| Extrusion/Intrusion, mm | - | 0.1 E | 0.6 E | 0.3 E | 0.3 I | 0.6 E | 0.3 E | 0.1 E | 0 | 0.1 E | 1.6 E | 2.1 E | - | - | 0.4 E | 0.4 E |
| Relative Ext/Int, mm | - | - | - | - | - | - | - | - | - | - | - | - | - | - | - | - |
| Translation Buccal/Lingual | - | 1.9 B | 1.8 B | 2.0 B | 1.3 B | 0 | 0.2 B | 0.4 B | 0.7 B | 1.3 B | 0.6 B | 0.3 B | - | - | 3.0 B | 3.2 B |
| Translation Mesial/Distal | - | 1.1 M | 1.9 M | 1.5 M | 0.8 M | 0.9 M | 3.8 D | 0.8 D | 0.3 M | 2.6 D | 1.1 M | 2.4 M | - | - | 1.0 M | 1.3 D |
| Rotation, ° | - | 1.0 M | 5.5 D | 1.5 D | 2.5 D | 0 | 6.8 M | 1.3 M | 3.9 M | 0.7 M | 0 | 11.0 D | - | - | 11.0 D | 0.3 M |
| Angulation, ° | - | 3.1 M | 5.6 M | 5.0 M | 2.4 M | 2.7 M | 11.0 D | 2.2 D | 0.8 M | 8.2 D | 1.4 M | 6.6 M | - | - | 3.9 M | 4.2 D |
| Inclination, ° | - | 6.4 B | 5.6 B | 5.2 B | 3.6 B | 1.0 L | 0.6 L | 1.1 B | 2.0 B | 2.4 B | 0.4 L | 0.9 L | - | - | 10.5 B | 11.4 B |

| **Lower Crown** | 48 | 47 | 46 | 45 | 44 | 43 | 42 | 41 | 31 | 32 | 33 | 34 | 35 | 36 | 37 | 38 |
| --- | --- | --- | --- | --- | --- | --- | --- | --- | --- | --- | --- | --- | --- | --- | --- | --- |
| Extrusion/Intrusion, mm | - | 0 | 0.1 E | 1.4 E | 0.2 I | 0 | 0.3 I | 0.6 I | 0.4 I | 0 | 0.3 E | 0.4 E | - | - | 0.5 E | 0.4 E |
| Relative Ext/Int, mm | - | 0.1 E | 0 | 1.4 E | 0.2 I | 0 | 0.2 I | 0.5 I | 0.3 I | 0.1 E | 0.2 E | 0.5 E | - | - | 0.5 E | 0.4 E |
| Translation Buccal/Lingual | - | 1.0 L | 0.5 L | 1.1 L | 0.6 L | 0 | 0.2 L | 0.3 L | 0.2 L | 0.2 L | 0.2 B | 0.2 L | - | - | 0.3 L | 0.5 L |
| Translation Mesial/Distal | - | 0 | 0.1 M | 0.3 M | 0.3 D | 0.3 D | 0.3 D | 0.2 D | 0.1 M | 0 | 0 | 0.2 D | - | - | 0 | 0.1 D |
| Rotation, ° | - | 0.8 D | 3.7 D | 49.4 D | 11.1 D | 3.7 M | 2.4 M | 0.5 M | 2.1 M | 4.7 M | 6.1 M | 0.3 M | - | - | 4.8 D | 1.1 D |
| Angulation, ° | - | 0.1 M | 3.7 D | 0.1 D | 3.5 D | 7.7 M | 2.7 M | 2.3 M | 1.9 M | 4.3 M | 12.4 M | 3.5 D | - | - | 4.2 D | 1.3 D |
| Inclination, ° | - | 5.8 L | 0.8 L | 0.5 L | 2.8 B | 0.3 B | 0.1 L | 1.9 B | 1.8 B | 0.2 L | 1.9 B | 1.1 L | - | - | 2.2 B | 1.2 L |

| **Lower Root** | 48 | 47 | 46 | 45 | 44 | 43 | 42 | 41 | 31 | 32 | 33 | 34 | 35 | 36 | 37 | 38 |
| --- | --- | --- | --- | --- | --- | --- | --- | --- | --- | --- | --- | --- | --- | --- | --- | --- |
| Extrusion/Intrusion, mm | - | 0 | 0.1 E | 1.4 E | 0.2 I | 0 | 0.3 I | 0.6 I | 0.4 I | 0 | 0.3 E | 0.4 E | - | - | 0.5 E | 0.4 E |
| Relative Ext/Int, mm | - | - | - | - | - | - | - | - | - | - | - | - | - | - | - | - |
| Translation Buccal/Lingual | - | 0.7 B | 0.2 L | 0.9 L | 1.4 L | 0.1 L | 0.2 L | 0.8 L | 0.8 L | 0.1 L | 0.5 L | 0.1 B | - | - | 0.9 L | 0.2 L |
| Translation Mesial/Distal | - | 0.1 D | 1.2 M | 0.3 M | 0.8 M | 3.4 D | 1.1 D | 0.9 D | 0.5 D | 1.4 D | 4.8 D | 0.8 M | - | - | 1.2 M | 0.3 M |
| Rotation, ° | - | 0.8 D | 3.7 D | 49.4 D | 11.1 D | 3.7 M | 2.4 M | 0.5 M | 2.1 M | 4.7 M | 6.1 M | 0.3 M | - | - | 4.8 D | 1.1 D |
| Angulation, ° | - | 0.1 D | 3.7 M | 0.1 M | 3.5 M | 7.7 D | 2.7 D | 2.3 D | 1.9 D | 4.3 D | 12.4 D | 3.5 M | - | - | 4.2 M | 1.3 M |
| Inclination, ° | - | 5.8 B | 0.8 B | 0.5 B | 2.8 L | 0.3 L | 0.1 B | 1.9 L | 1.8 L | 0.2 B | 1.9 L | 1.1 B | - | - | 2.2 L | 1.2 B |
